# Motility and Ultrastructure of *Spirochaeta thermophila*

**DOI:** 10.3389/fmicb.2016.01609

**Published:** 2016-10-14

**Authors:** Reinhard Wirth, Matthias Ugele, Gerhard Wanner

**Affiliations:** ^1^Faculty of Biology, University of RegensburgRegensburg, Germany; ^2^In-Vitro DX and Bioscience, Department of Strategy and Innovation, Siemens Healthcare GmbHErlangen, Germany; ^3^Department of Biology I, Ludwig-Maximilian-UniversityMunich, Germany

**Keywords:** *Spirochaeta thermophila*, cell body plan, changing cell morphology, motility, high temperature light microscopy, electron microscopy

## Abstract

We analyze here for the first time the swimming behavior of a thermophilic, strictly anaerobic Spirochete, namely *Spirochaeta thermophila* using high temperature light microscopy. Our data show that *S. thermophila* very rapidly can change its morphology during swimming, resulting in cells appearing nearly linear, in cells possessing three different spiral forms, and in cells being linear at one end and spiral at the other end. In addition cells can rapidly bend by up to 180°, with their ends coming into close contact. We combine electron with light microscopy to explain these various cell morphologies. Swimming speeds for cells with the various morphologies did not differ significantly: the average speed was 33 (± 8) μm/s, with minimal and maximal speeds of 19 and 59 μm/s, respectively. Addition of gelling agents like polyvinylpyrrolidone or methyl cellulose to the growth medium resulted in lower and not higher swimming speeds, arguing against the idea that the highly unusual cell body plan of *S. thermophila* enables cells to swim more efficiently in gel-like habitats.

## Introduction

In 1676 Antoni van Leeuwenhoek discovered microorganisms using his own-made single-lens microscope. His self-fabricated biconvex or planoconvex lenses could resolve structures of 700 nm; this resolution has been proven by taking pictures through an original Leeuwenhoek microscope (Ford, 2011). Over the centuries light microscopy has been optimized, e.g., by use of two-lens microscopes, introduction of phase-contrast microscopy and developed into new and very special fields (for reviews see e.g., Keller et al., 2009; Kasper and Huang, 2011; Coltharp and Xiao, 2012). In addition, a combination of various microscopic techniques can be used to obtain high specificity and detailed structural information (Smith, 2012). Special techniques today overcome the resolution limit (~200 nm) of light microscopy (Keller et al., 2009; Kasper and Huang, 2011). This is especially true for electron microscopy (EM) with its various disciplines like transmission EM (TEM), scanning EM (SEM), cryo EM, focused ion beam EM, etc. Modern techniques allow e.g., high-resolution EM of cellular components, initially with a resolution of ~7 Å (Yu et al., 2012), and today even better, down to near atomic resolution of ~4 Å (e.g., Bernecky et al., 2016; Braun et al., 2016; Taylor et al., 2016; Zoued et al., 2016). Even whole small bacterial cells meanwhile have been analyzed with a resolution of ~4 Å (Oikonomou and Jensen, 2016).

**Figure 1:**
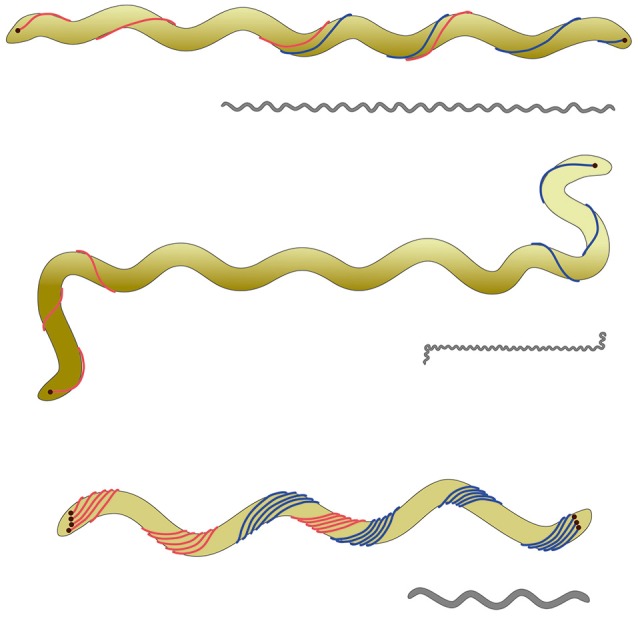
**Scheme of the cell body plan of various spirochetes**. The clolored scheme shows from top to bottom *Spirochaeta thermophila, Leptospira illini*, and *Borrelia burgdorferi*. For all three cells the outer membrane—enclosing the endoflagella—is not shown for the sake of clarity. In the case of *S. thermophila* the endoflagella overlap in a 1:2:1 arrangement; the endoflagella of *L. illini* are too short to overlap in the middle. In the case of *B. burgdorferi* the endoflagella form a ribbon, wrapping around the cylindrical cell body. It has to be emphasized that *L. illini* and *S. thermophila* have a spiral shape, whilst *B. burgdorferi* comes as a flat wave. The gray drawings give an impression of the proportions of these three cells with an average length of 20, 10, and 10 μm for *S. thermophila, L. illini*, and *B. burgdorferi*, respectively.

**Figure 2:**
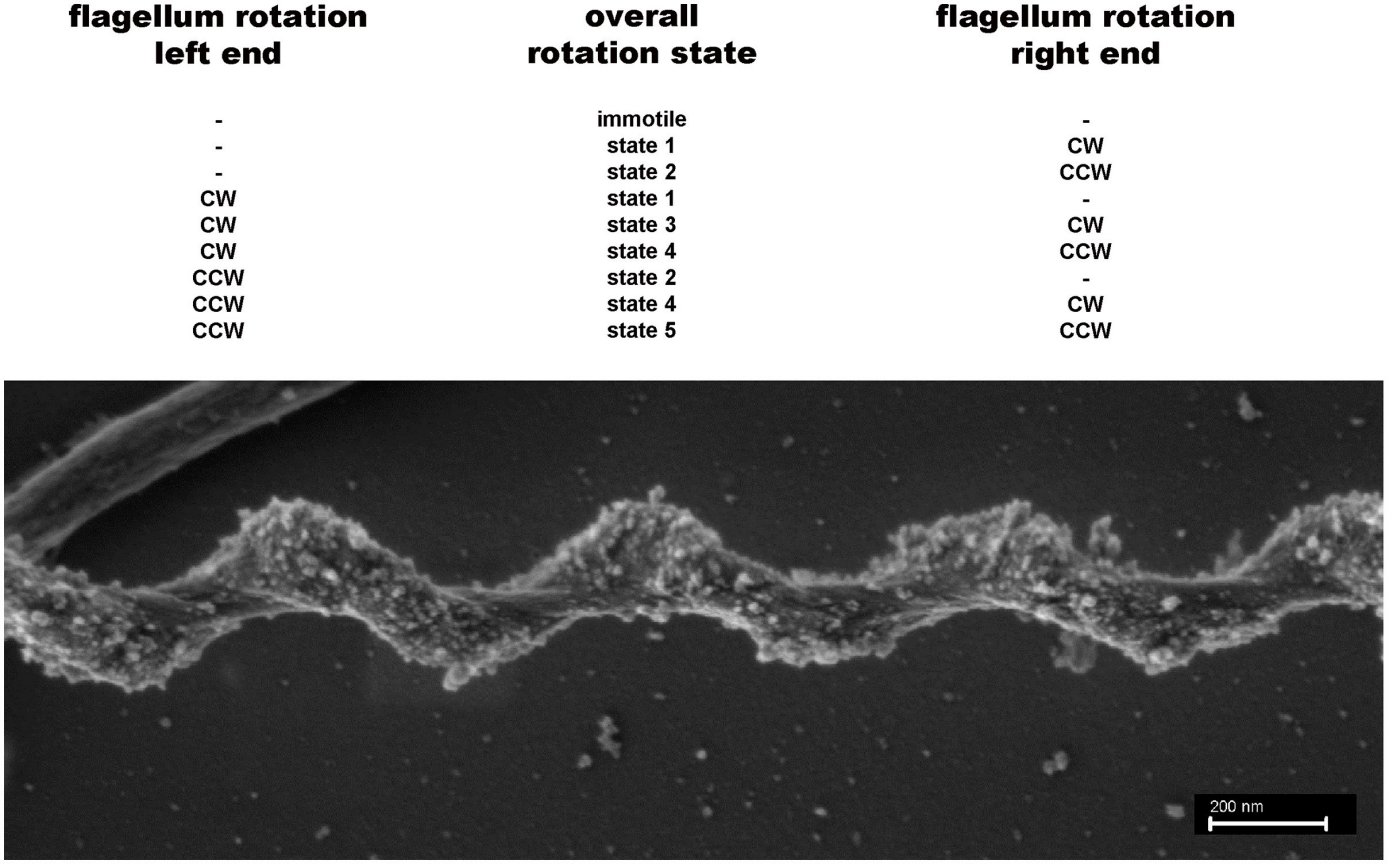
**Potential different rotation states for endoflagella of spirochetes**. Top: A total of 9 different rotations states (none = −; clockwise = cw; counterclockwise = ccw) for the flagella inserted on the left and right poles of the spiral shaped cells are possible. If the left and right ends of the cells are functionally indistinguishable 5 rotation states remain. States 3 and 5 probably are different, because a cw rotation of flagella should exert a different force on the spiral shaped cell body compared with a ccw flagellar rotation. Bottom: SEM picture of a *S. thermophila* cell, clearly showing that the spiral-shaped cell body winds around the endoflagella, which themselves are covered by the outer sheath.

Spirochetes are—due to their very special cell body plan—a fascinating group of bacteria; most interestingly they can swim by use of so-called endoflagella. These endoflagella are located between the cell body and the outer membrane, i.e., in the periplasmic space of the Gram-negative bacteria. Flagella are inserted into the cell body at both ends of a cell, they can overlap in the middle region—if only one flagellum is inserted per cell pole one speaks of a 1:2:1(end:middle:end) flagellar arrangement (Leschine et al., 2006). In some cases, like *Leptonema illini*, the endoflagella are too short to overlap; those cells have, depending on their motility status, hook-shaped ends, (for further details see e.g., **Figures 2**, **3** in Wolgemuth, 2015). Spirochetes come in two different shapes: either a helix, like e.g., *L. illini* (Wolgemuth, 2015) or a flat wave, like e.g., *Borellia burgdorferi* (Charon et al., 2012). A scheme on the cell body plan of *B. burgdorferi, L. illini*, and *Spirochaeta thermophila*—the subject of this study—is given in Figure 1.

*S. thermophila* is a member of the phylum *Spirochaetes* which contains important disease causing species like *Treponema pallidum* (syphilis), *B. burgdorferi* (lyme disease), and *Leptospira interrogans* (leptospirosis, resulting in renal failure). Recently a phylogenomic analysis identified a 3 amino acid conserved signature indel in the FlgC protein as a unique molecular marker for this phylum, resulting in a proposal for a taxonomic revision (Gupta et al., 2013). The genus *Spirochaeta* is comprised of anaerobic and facultative aerobic spirochetes that are indigenous to aquatic environments. All cultivated species of the genus *Spirochaeta* are mesophiles with the exception of *Spirochaeta caldaria* and *Spirochaeta thermophila* having optimal growth temperatures of 48–52°C and 66–68°C, respectively. For mesophilic spirochetes, especially pathogenic ones like *L. illini* (Goldstein et al., 1996) and *B. burgdorferi* many data are available, including e.g., the structure and assembly of the endoflagella (Zhao et al., 2013, and references therein), or their mode of motility (Goldstein et al., 1994; Motaleb et al., 2000; Charon et al., 2012). To the best of our knowledge, however, no data as to the motility of any thermophilic spirochete is available.

In many cases members of the phylum *Spirochaetes* are found in habitats characterized by an increased viscosity like mucous membranes. It has been reported that swimming speed increases if such spirochetes are transferred from aqueous media to more viscous ones (see Canale-Parola, 1978 for details). This was taken as explanation for the unique cell body plan of spirochetes with their flagella contained within the outer sheath of the cell body. The argument is that a screw-like movement of the spiral shaped bacteria through highly viscous media like slime overlying mucous membranes should be superior to the attempt to move bacteria in viscous environments by rotation of extracellular flagella.

The very unusual cell body plan of spirochetes has attracted much attention and various hypotheses upon their motility mechanisms have been formulated, see e.g., (Canale-Parola, 1978). The most popular one, namely Berg's model for spirochetal motility states that the endoflagella inserted at the cell poles do rotate and cause counter-rotation of the helical protoplasmic cylinder within the outer sheath. Since flagella can rotate clockwise or counterclockwise or not at all, one can state that a total of 9 different rotation states should be possible. Of those only one—namely no rotation at both cell poles—should result in immotile cells, whilst the 8 other potential rotation states should result in at least four, but very probably five different functional rotation states. Figure 2 characterizes these functional states, which are based on the assumption that the left and right ends of the spiral shaped cells are functionally equivalent.

We analyzed here the swimming behavior of *S. thermophila*, using a newly developed device (Mora et al., 2014) which can be added onto any upright light microscope to allow analyses at high temperatures under anaerobic conditions. Our data prove the extraordinary flexibility of the cells during swimming, with rapid changes between linear cells, cells possessing at least three different spiral forms and flexing cells, bending by 180°. Our combination of TEM, SEM, and light microscopy studies gives a hint how the various cell morphologies can be correlated with the various flagellar rotation states possible for this spirochete.

## Materials and methods

### Microorganisms and growth conditions

*S. thermophila* (DSMZ strain 6192) was grown anaerobically at 65°C in a self-developed medium, based on medium 509 by DSMZ (Deutsche Sammlung für Mikroorganismen und Zellkulturen; Braunschweig, Germany). Instead of preparing the medium anaerobically from 7 different, sterile filtered components—as recommended by DSMZ—the following procedure was used. In 995 ml distilled water were dissolved: 4 g NaCl; 1 g MOPS; 0.8 g MgCl_2_ × 6 H_2_O; 0.5 g KCl; 0.3 g NH_4_Cl; 0.2 g KH_2_PO_4_; 0.03 g CaCl_2_ × 2 H_2_O; 1 mg resazurin; 1 ml Wolfe's Vitamins (Huber and Stetter, 2006); 1 ml Trace Mineral Solution (Huber and Stetter, 2006). The medium was bubbled with N_2_/CO_2_ for 30 min, and reduced by addition of 1 ml H_2_O containing 0.15 g Na_2_S × 3 H_2_O. After adjusting the pH anaerobically to 6.8 with 1M NaOH, 20 ml were aliquoted in an anaerobic chamber into 120 ml serum bottles, the gas phase readjusted to N_2_/CO_2_ (80:20%), followed by autoclaving. For use the serum bottles were supplemented with 1 ml anaerobically prepared 5% NaHCO_3_ plus 0.2 ml anaerobically prepared 40% glucose. This modified medium allowed growth to an optical density of ca. 0.3, a cell density already too high for taking videos.

### High-temperature light microscopy

The original thermomicroscope (Horn et al., 1999) including data acquisition and handling was used as described earlier (Herzog and Wirth, 2012) for initial experiments. In short, cells were transferred to rectangular glass capillaries, which were closed by super glue and analyzed using a specially modified Olympus BX50 microscope.

**Figure 3:**
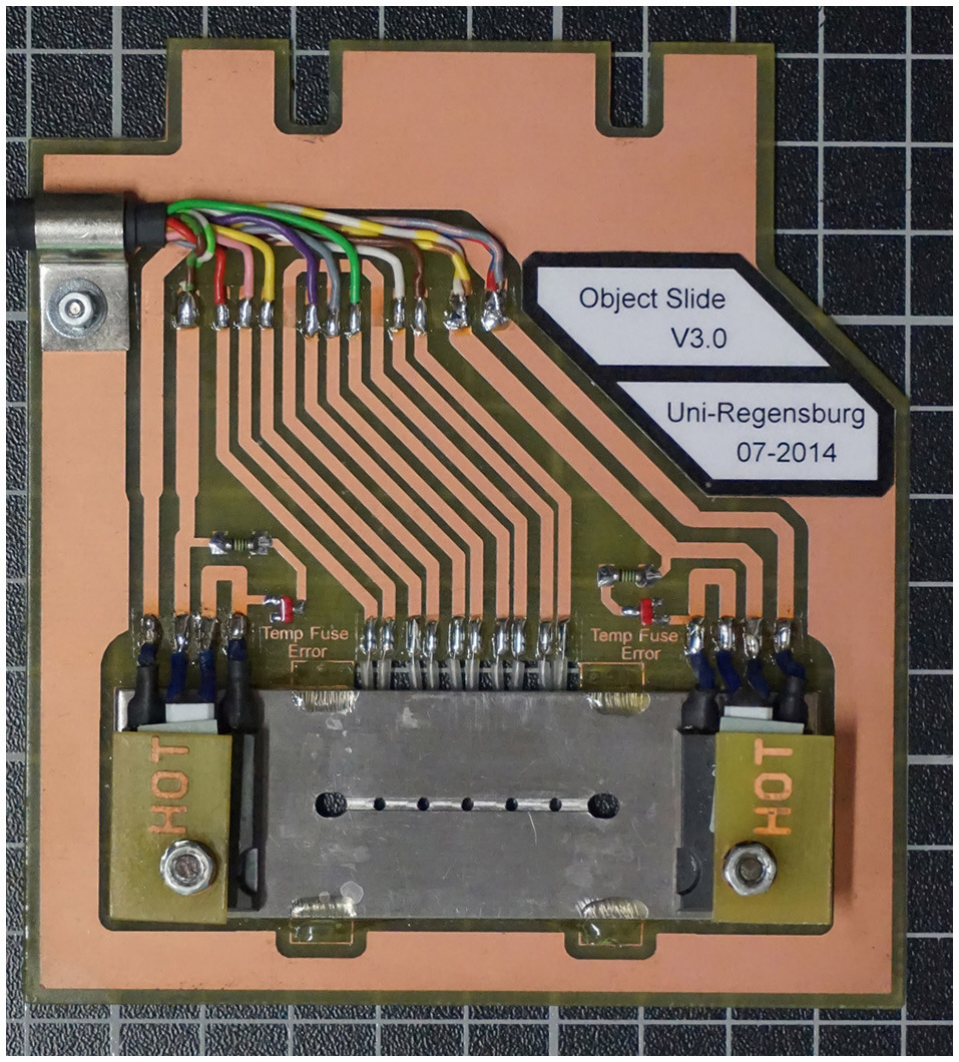
**The temperature gradient forming device TGFD**. A stainless steel plate contains a rectangular groove with five observation holes (total distance 2 cm). Into this groove a rectangular glass capillary (inner height 100 μm) containing the cell suspension is placed. The 3 cm long glass capillary is closed on both sides with instant glue (two outermost bigger holes). Two heating elements (HOT) allow a temperature gradient of > 40°C to be established; the actual temperature is measured via five heat sensors at the backside of the circuit board holding the stainless steel plate (see Mora et al., 2014 for details).

Most of our analyses, however, were done by the use of a new low-budget device, which we developed in cooperation with the electronic workshop of the Faculty for Biology of the University of Regensburg. It is called TGFD (temperature gradient forming device), because it allows establishing a temperature gradient of over 40°C at a distance of just 2 cm within a few minutes; Figure 3 shows the latest version of the TGFD. It allows microscopy under anaerobic conditions at temperatures of up to 110°C. This device can mimic temperature gradients found in the natural habitat of e.g., hyperthermophiles; details are given in Mora et al. (2014).

Movies were recorded using a pco1600 camera (2 GB intern memory) and analyzed using open source software ImageJ (version 1.410) with add-on modules particle tracker and manual tracking. Tracks of at least 20 consecutive frames were used for calculations. The speed of 3 to 10 different cells from one sample was averaged and experiments were run in 3-fold repetitions, to reduce experimental variations.

### Electron microscopy

We used established procedures for electron microscopic investigations (Rachel et al., 2010; Jogler et al., 2011). These included: negative staining for analyses of cell shape; thin sectioning and TEM to ask for the number of endoflagella; SEM to analyse the morphology of *S. thermophila*.

## Results

### The cell body plan of *S. thermophila*

**Figure 4:**
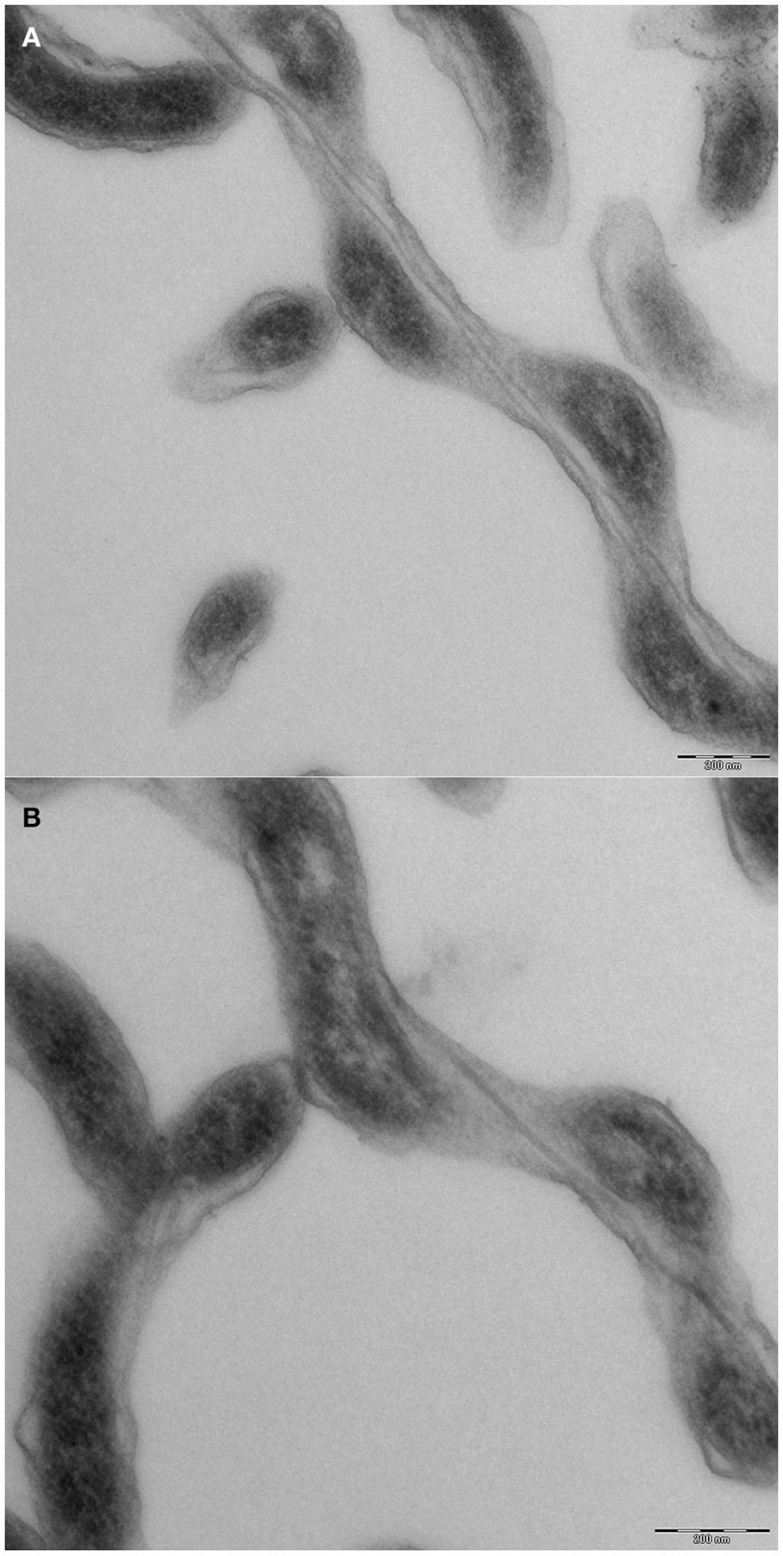
**Cell morphology of ***Spirochaeata thermophile***—TEM analyses of thin sections**. Cells of *S. thermophila* were grown to late exponential phase and fixed by addition of EM grade glutardialdehyde to an end concentration of 2%. Further processing included negative contrasting and thin sectioning. **(A)** middle part of a cell, clearly showing two endoflagella; **(B)** end of a cell, with only one endoflagellum.

We were interested in the swimming behavior of *S. thermophila*, because spirochetes generally are described to be extremely flexible bacteria and their cell body plan differs from all other motile bacteria (Leschine et al., 2006). It has to be noted, however, that not a single body plan of spirochetes exists: it has been shown that *B. burgdorferi* is swimming in form of a flat wave (Goldstein et al., 1994; Motaleb et al., 2000; see Charon et al., 2012 and Wolgemuth, 2015 for detailed discussions), whilst *L. illini* was reported to “literally screw through a gel-like medium such as 1% methylcellulose” (Goldstein et al., 1996). Our TEM analyses (see Figure 4) confirmed that *S. thermophila* has a single endoflagellum inserted at each pole to overlap in the middle of the cell (1:2:1 flagellar arrangement). The cells are considerably long and slender, with an average length of ca. 25 μm (maximum length of 60 μm) and have a diameter of ca. 0.23 μm. These findings were supported by light microscopy and are characteristic for the genus *Spirochaeta* (Leschine et al., 2006). *S. thermophila* cells cleary do not show a flat wave morphology, they rather are spiral-shaped (see e.g., Figure 2).

### *Spirochaeta thermophila* is an extremely flexible thermophilic bacterium which can adopt various wave forms during swimming

**Figure 5:**
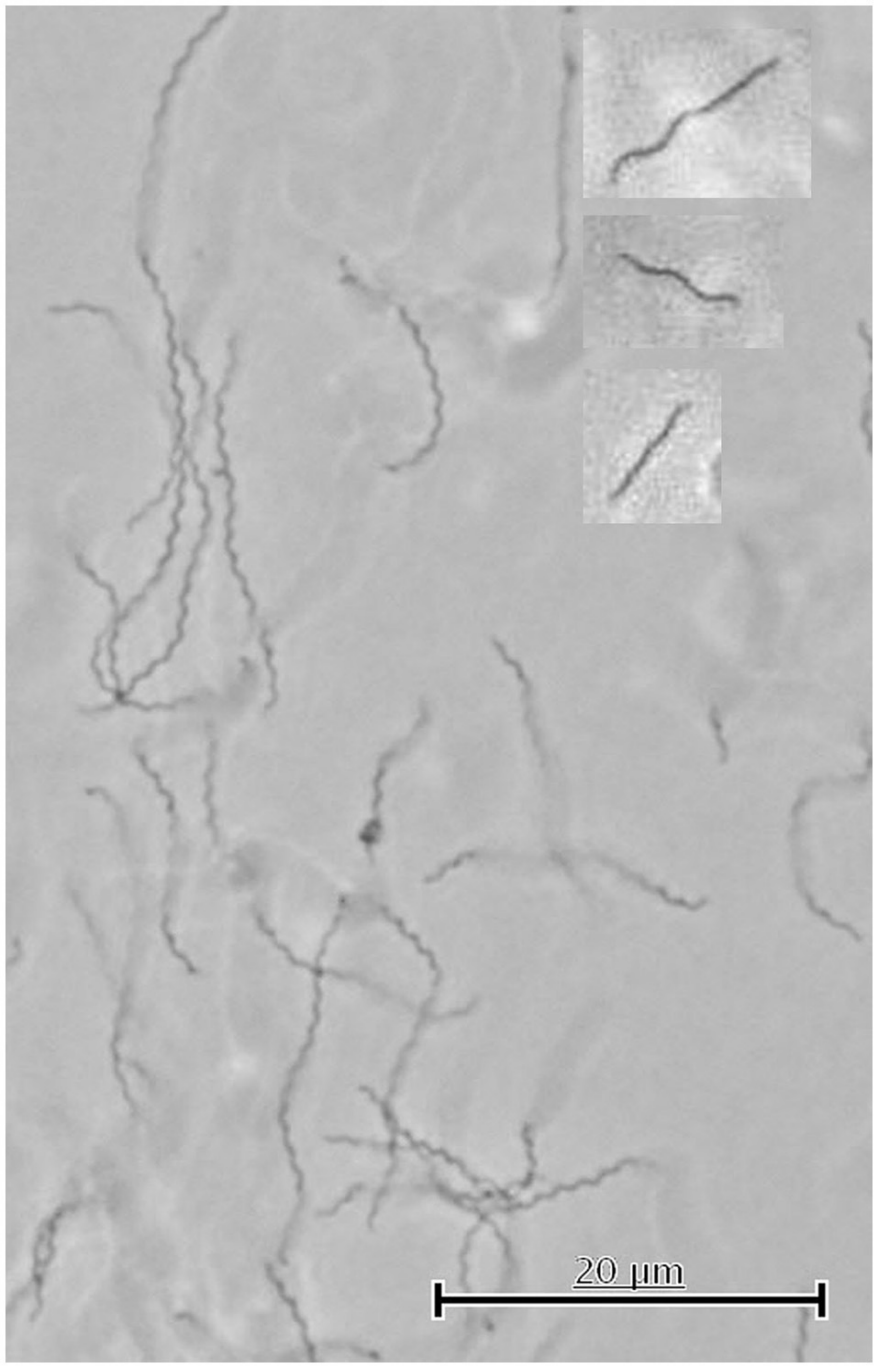
**Cell shapes of immotile and swimming ***S. thermophila*** cells observed by light microscopy**. This picture shows immotile cells as observed by light microscopy done aerobically at room-temperature. The cell shapes of swimming cells (analyzed by light microscopy at 65°C and anaerobically) indicated in the text by (1), (2), and (3) are shown in the insets from top to bottom. Note that light microscopy cannot differentiate between linear and slightly spiral-shaped cells (see Figure 4, cells [d] and [b]) and therefore 5 different cell shapes of swimming cells occur.

**Figure 6:**
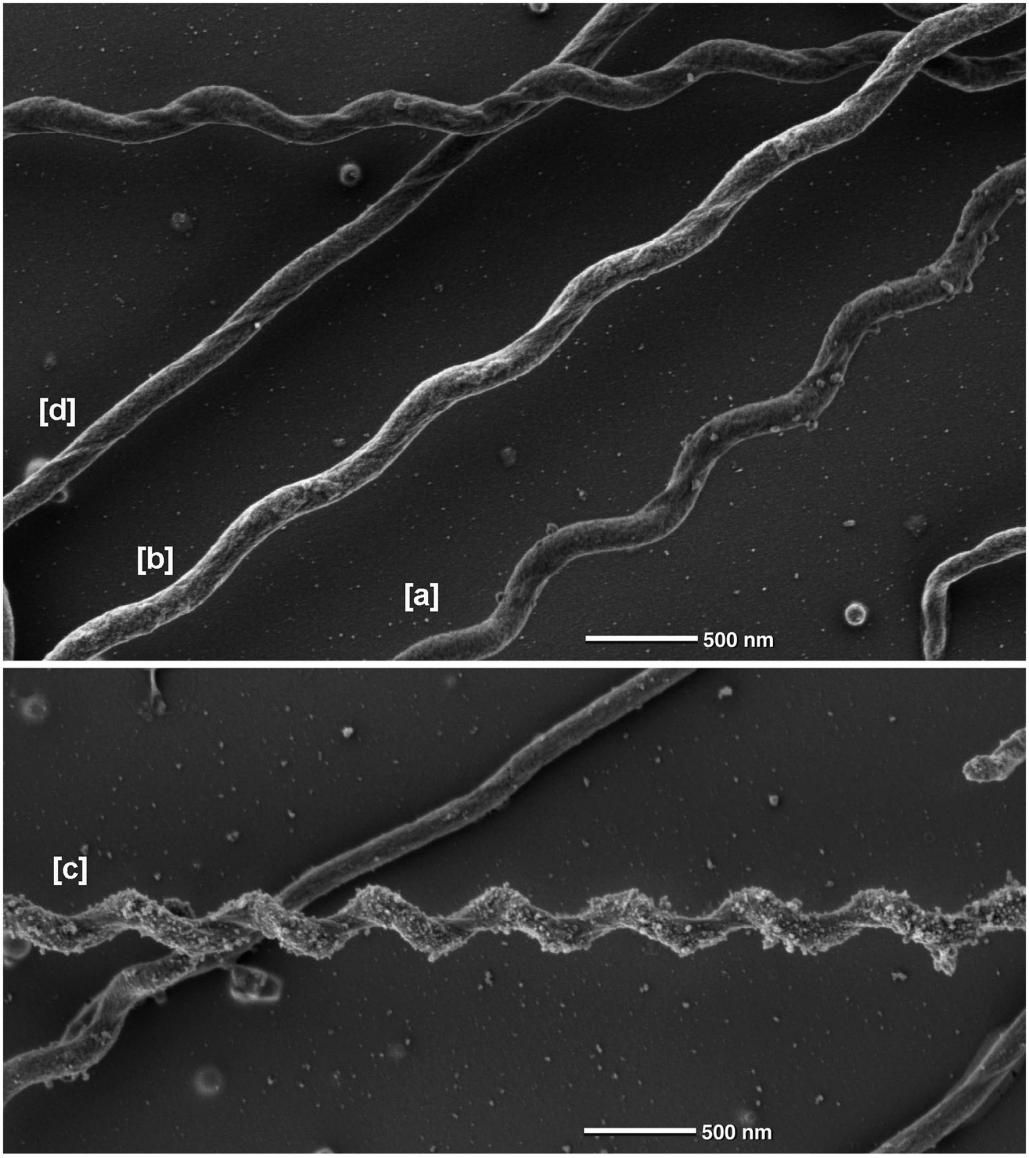
**Different morphological forms of ***Spirochaeta thermophila*** observed by scanning electron microscopy**. Data were obtained by SEM. The cells indicated by [a], [b], and [c] have a wave-length of 0.88, 0.91, 0.56 μm and wave-amplitudes of 0.26, 0.22, 0.29 μm, respectively; [d] shows a linear cell.

We used various approaches to ask for the morphology of *S. thermophila*:

- Light microscopy of cells under non-physiological conditions—i.e., at room-temperature and aerobic conditions—indicated that results obtained under such conditions are artificial. All cells were immotile and exhibited only one waveform, namely ca. 1.25 × 0.25 μm (wave length × amplitude—see Figure 5) after 15 min of collection from freshly grown cultures. These immotile cells are supposed to represent the cell shape if flagella are not rotating. If cells were observed within 2 min after collection a few of them (<3%) exhibited other forms than those described in (ii) with wave lengths of up to 1.2 μm and amplitudes of up to 0.6 μm; after 15 min, however, essentially all cells had the waveform of 1.25 × 0.25 μm. These same cells (grown anaerobically at 65°C) showed good motility if analyzed as described in (iii). We give here the dimensions in wave length × amplitude, although helix pitch and diameter are more correct for a spiral shaped cell; the wave length × amplitude dimensions are commonly used by others regardless if cells come as helices or flat waves.
- SEM analyses, in contrast to the results of (i), indicated the existence of three different spiral forms—see Figure 6: cells with a wave-length of ca. 0.88 μm and an amplitude of 0.26 [a] μm make up the majority (ca. 60%), whilst cells with 0.94 × 0.22 μm [b] and those with 0.56 × 0.29 μm [c] make up less than 10%. In addition we observed approximately linear cells [d]. We finally identified also linear cells which sometimes converted at one end into the most prominent waveform (see Supplemental Movie 2). Examples for the forms [a] to [d] observed by SEM are shown in Figure 6. The SEM data also proved that *S. thermophila* cells do not exhibit flat wave morphology, but rather have a helical shape (Figures 2, 6). This is also supported by our movies: swimming cells do not exhibit the rapid periodically transition between wave form and straight appearance, which is characteristic for a rotating flat wave and was observed e.g., in the case of *B. burgdorferi* (see Supplemental Movie 1 in Charon et al., 2012). In addition, some cells constantly show a spiral shape during swimming—see Supplemental Movie 3.
- High-temperature light microscopy under anaerobic conditions revealed striking differences to light microscopic data obtained under non-physiological conditions [aerobiosis and room-temperature—see (i), above]. Most obvious, of course, was the ability of cells to swim; in addition, the cells changed rapidly between different waveforms during swimming; for an overview, see Supplemental Movie 1. We identified swimming cells with the following cell shapes: (1) one half of the cell has a spiral-like shape, whilst the other half appears as approximately straight; (2) the cell has a spiral-like shape over its total length; (3) the cell appears over its total length as approximately straight line; these three shapes are exemplified by the insets in Figure 5. Supplemental Movies 2–4 exemplify these various morphological forms during swimming, as observed by light microscopy. It has to be emphasized that these forms are from live cells, whilst aerobic observation at room-temperature resulted completely in the waveform (1.25 × 0.25 μm) of immotile cells, mentioned above. We also note that the resolution of light microscopy is not able to differentiate between different shapes of cells appearing as approximately straight lines which we could differentiate by electron microscopy—i.e., the cells labeled [d] and [b] in Figure 6. Therefore, we could characterize in total five different shapes of swimming *S. thermophila* cells, which very well might reflect the five different endoflagella rotation states outlined in Figure 2. Our motility analyses showed that *S. thermophila* cells can be very flexible, in extreme cases they bend to at least 180° during swimming, or sometimes even come close to form “knot-like” structures; Supplemental Movie 5 highlights such observations. The cell bend within 200 ms to 180° (=6 frames of the movie taken at 32 fps) as shown in Supplemental Movie 5. These data indicate that the earlier described flexing motility for other spirochetes (see Canale-Parola, 1978 for details) also can be observed in *S. thermophila*.

Our experiments gave no indication for the ability of *S. thermophila* to swim faster by adding polyvinylpyrrolidone (PVP) as gelling agent to the medium. PVP in end concentrations of up to 2.0% did not influence growth of *S. thermophila*, whilst 3% PVP resulted in retarded growth. Indeed, the addition of PVP (in concentrations of 0.5; 1.0; 1.5 and 2.0%) did not enhance, but rather decreased both the percentage of motile cells and their swimming speed from 33 to < 1 μm/s. Similar data were obtained with methyl cellulose (MC) as gelling agent—in this case, an end concentration of 1.0% already had a weak growth inhibitory effect. We concluded that in the case of *S. thermophila* swimming speed decreases with increasing concentration of gelling agents—see also discussion below.

## Discussion

The data obtained here and earlier (Herzog and Wirth, 2012; Mora et al., 2014) clearly show that statements on the motility of anaerobic (hyper) thermophilic microorganisms cannot be made if cells are analyzed under non-physiological conditions (=aerobic light microscopy at room-temperature). In the case of *S. thermophila* light microscopy under such conditions results in the existence of immotile cells of primarily one morphotype—this very clearly is an artifact.

Our swimming movies indicated the existence of at least three different cell morphologies; in combination with SEM analyses even five different cell morphologies could be identified: cells being approximately linear over their total length; cells exhibiting a half-linear half-spiral form; and cells possessing one of three different spiral forms. Very interestingly the cells can rapidly switch between these different morphotypes during swimming. At the moment it would be pure speculation which of the five different cell morphologies corresponds with the five different endoflagella rotation states outlined in Figure 2. As mentioned above, the 1.25 × 0.25 μm (wave length × amplitude) cells very probably represent the state in which both flagella are not rotating, i.e., the immotile state of Figure 2.

Our data show that *S. thermophila* exhibits a spiral form; its cell body plan therefore does not resemble *B. burgdorferi* which has flat wave morphology. On the other hand *S. thermophila* also does not resemble *L. illini* in its cell body plan, because the endoflagella of *S. thermophila* exhibit a 1:2:1 arrangement, i.e., they overlap in the middle of the cell. In addition we never observed the hook-like ends, being characteristic of *L. illini* or *L. interrogans* (Wolgemuth, 2015). It was brought to our attention that in theory one might be able to estimate the rotational rate of the cell using the cell's shape and their swimming speed. One even might be able to predict the cell stiffness; for such calculations certain assumptions (like: swimming speed is determined mainly by rotation of the helical cell body) have to be made about which we feel not sure. Therefore, these theoretical calculations are not included here. We want to stress, however, the fact that the morphotype of spirochetes well can change. In the case of *B. burgdorferi* the flat wave form of the cells alters to a rod form, if the endoflagella are not expressed (Motaleb et al., 2000), or the motor does not rotate (Sultan et al., 2015). In the case of *Treponema denticola* with a 2:4:2 endoflagella arrangement an irregular twisted morphology bas been observed. Both planar and helical regions of the cells were observed with endoflagella wrapped around planar regions or lying along the cell axis in helical regions. In addition, however, also some *T. denticola* cells with completely right-handed morphology have been observed (Ruby et al., 1997). All these data point to the fact that only a combination of light microscopy (done under physiological conditions to observe motility) with electron microscopy—as we have done here—will allow one to define the cell body plan of spirochetes.

The addition of PVP or MC as gelling agent in our experiments led to a decrease in the swimming speed of *S. thermophila*; these agents, however not only increase the viscosity, but also elasticity. Interestingly, an increase in viscosity alone—by addition of Ficoll—slowed down, both *B. burgdorferi* and *T. pallidum* (Harman et al., 2013). On the other hand the swimming speed of *B. burgdorferi* did increase by an increase of viscoelasticity via the addition of 1% MC from <1 μm/s to ~10 μm/s (Li et al., 2010). Similar viscoelasticity effects were reported for *T. denticola* (Ruby and Charon, 1998). We therefore conclude that *S. thermophila* does not react to a gel-like habitat like the pathogenic spirochetes do by altering their swimming speed. The normal habitat of *S. thermophila* indeed, is not characterized by marked viscosity or viscoelasticity: the type strain was isolated from a marine hot spring in Kamchatka (Aksenova et al., 1992).

## Author contributions

RW planned the study, took some of the movies and wrote the manuscript. MU took most of the movies. GW made all electron microscopic pictures.

### Conflict of interest statement

The authors declare that the research was conducted in the absence of any commercial or financial relationships that could be construed as a potential conflict of interest.

## Supplementary material

Supplemental Movie 1 **Swimming of *Spirochaeta thermophila* at 65°C—overview**.

Supplemental Movie 2 **Swimming of *Spirochaeta thermophila* at 65°C; detail to indicate cell shape (1) for a cell swimming “upwards”—one half of the cell has a spiral-like shape, whilst the other half appears as approximately straight**.

Supplemental Movie 3 **Swimming of *Spirochaeta thermophila* at 65°C; detail to indicate cell shape (2) for a cell moving from left to right and reversing—the cell has a spiral-like shape over its total length**.

Supplemental Movie 4 **Swimming of *Spirochaeta thermophila* at 65°C; detail to indicate cell shape (3) for a cell moving from top right to bottom left—the cell appears over its total length as approximately straight line**.

Supplemental Movie 5 **Swimming of *Spirochaeta thermophila* at 65°C; detail to indicate very rapid flexing of cells—see text for further explanation**.
